# Comprehensive and computational analysis of genes in human umbilical vein endothelial cells responsive to X-irradiation

**DOI:** 10.1016/j.gdata.2016.05.007

**Published:** 2016-05-16

**Authors:** Yukihiro Furusawa, Qing-Li Zhao, Yuichi Hattori, Yoshiaki Tabuchi, Toshiyasu Iwasaki, Takaharu Nomura, Takashi Kondo

**Affiliations:** a Department of Radiological Sciences, Graduate School of Medicine and Pharmaceutical Sciences, University of Toyama, 2630 Sugitani, Toyama 930-0194, Japan; b Department of Liberal Arts and Sciences, Toyama Prefectural University, 5180 Kurokawa, Toyama 939-0398, Japan; c Department of Molecular and Medical Pharmacology, Graduate School of Medicine and Pharmaceutical Sciences, University of Toyama, 2630 Sugitani, Toyama 930-0194, Japan; d Division of Molecular Genetic Research, Life Science Research Center, University of Toyama, 2630 Sugitani, Toyama 930-0194, Japan; e Radiation Safety Research Center, Nuclear Technology Research Laboratory, Central Research Institute of Electric Power Industry, 2-11-1 Iwado Kita, Komae, Tokyo 201-8511, Japan

**Keywords:** Ionizing radiation, Cardiovascular disease, Human umbilical endothelial cells, Inflammatory response, Microarray

## Abstract

Radiation exposure such as A-bomb or radiation therapy is considered a major health-risk factor for cardiovascular disease. In order to understand the molecular mechanisms underlying the inflammatory reaction frequently encountered in the vascular system after exposure to ionizing radiation, we carried out a global scale microarray and computational gene expression analyses on human umbilical endothelial cells (HUVECs) exposed to X-ray (2.5 Gy). The gene ontology analysis revealed that the down-regulated genes were associated with cell cycle regulation, whereas the up-regulated genes were associated with inflammatory responses, in particular, the type 1 interferon response. The computational analysis using ingenuity pathway analysis also identified a gene network containing the interferon response factor 7 (IRF7) and its transcriptional targets such as interferon-induced transcripts (IFITs) and Mx1, which have been known to be associated with inflammation in endothelial cells. The up-regulated genes and the gene network identified here may explain the inflammatory response induced by X-irradiation. These findings uncover part of the molecular basis of the mechanism(s) of the inflammatory disorder in response to X-irradiation in HUVECs. The dataset is publicly available at the Gene Expression Omnibus (GEO) repository (http://www.ncbi.nlm.nih.gov/geo/) with accession number GSE76484.

**Table:** 

Specifications	
Organism/cell line/tissue	Human umbilical vein endothelial cells (HUVECs)
Sex	n/a
Sequencer or array type	GeneChip® Human Genome U133 Plus 2.0 Array
Data format	Raw and processed
Experimental factors	Cells
Experimental features	Cells were irradiated with X-rays at a dose of 2.5 Gy and were harvested 6, 12, and 24 h after irradiation.
Consent	n/a
Sample source location	n/a

## 1 Direct link to deposited data

The microarray data was deposited in the Gene Expression Omnibus (GEO): http://www.ncbi.nlm.nih.gov/geo/query/acc.cgi?acc=GSE76484.

## 2 Introduction

Recently, the association of radiation with cardiovascular disease mortality in the life span study cohort of 86,000 A-bomb survivors with estimated doses was reported [1]. Radiation exposure, not only for A-bomb survivors, but also for patients with radiotherapy for cancer, has been considered as a major health-risk factor for cardiovascular disease [2], [3], despite the usefulness of radiation for clinical cancer therapy. Although chronically produced reactive oxygen species and inflammation are thought to be a pathogenic mediator of atherosclerosis [4], the detailed molecular mechanism of these events has remained unclear.

In a previous study, we addressed the effect of X-irradiation response on endothelial NO synthase (eNOS) expression and activation [5], which is considered to play a pivotal role in the inflammatory response. Endothelial cells are known to be highly sensitive to ionizing radiation and we showed the down-regulation of eNOS expression in rabbit ear central artery 1–4 weeks after X-irradiation at a relatively high dose (45 Gy) [6], probably due to the damage of endothelial cells. On the contrary, an increase in NO production was observed in human umbilical endothelial cells (HUVECs) at 6 h after X-irradiation at a dose of 2–10 Gy [5]. eNOS activation, but not induction, was observed 6–72 h, after exposure to 10 Gy X-rays, and NO levels reached a maximum at 72 h.

The contradiction of the radiation-induced changes in NO production may be explained by the differences of materials for assay, radiation dose, or stage (within a few days or 1–4 weeks later). The mechanisms by which the X-irradiation affects inflammatory response in HUVECs appear to be complex; thus, it remains to be further elucidated. In addition, we should further disclose the mechanisms, particularly beyond the perspective of eNOS expression and activation.

Recent microarray technology coupled with bioinformatics tools has provided a view of genome-wide expression profiles, as well as the relevant biological function and gene networks based on the gene-expression data [7]. Here, for better understanding the molecular mechanisms underlying the inflammatory reaction frequently encountered in vascular system after exposure to ionizing radiation, we carried out global scale microarray and computational gene expression analyses in HUVECs after X-irradiation by 2.5 Gy, which is similar to the dose for a fraction frequently used in clinical cancer treatment. In the present study, we focused on gene response associated with inflammation in HUVECs, in particular, at an early stage after irradiation.

## 3 Materials and methods

### 3.1 Cell culture and X-irradiation

HUVECs were cultured in Humedia EB-2 (Wako), as previously described [5]. In the present study, one million cells were seeded onto 60-mm culture dishes a day before irradiation. The cells were irradiated with X-rays at a dose of 2.5 Gy. X-ray irradiation at a dose rate of 5 Gy/min was performed using a Model MBR-1520R-3 X-ray unit (Hitachi Medico Technology, Kashiwa, Japan), as previously described [8], [9].

### 3.2 RNA isolation

The total RNA was extracted from cells using an RNeasy Total RNA Extraction kit (Qiagen, Valencia, CA) and treated with DNase I (RNase-free DNase kit, Qiagen) for 15 min at room temperature to remove residual genomic DNA. The RNA quality was analyzed using a Bioanalyzer 2100 and the RNA6000 Nano LabChip kit (Agilent Technologies, Inc., Santa Clara, CA). RNA samples with RNA integrity number (RIN) values above 9.0 were considered acceptable.

### 3.3 Microarray analysis

Microarray and computational gene expression analyses were performed using a GeneChip® system with a Human Genome U133-plus 2.0 array (Affymetrix, Santa Clara), which was spotted with approximately 54,000 probe sets, as previously described [9], [10], [11], [12]. Samples for array hybridization were prepared as described in the Affymetrix GeneChip® Expression Technical Manual. The scanned arrays were analyzed using the GeneChip Analysis Suite Software (Affymetrix). The obtained hybridization intensity data and qualities were checked using the GeneSpring® software.

### 3.4 Gene expression analysis

For global normalization, microarray signals were processed using a standard MAS5.0 algorithm [13]. Observed signals were normalized and genes that had no significant signals were ignored to reduce noise. In addition, probe sets targeting specific RefSeq transcripts based on RefDIC were extracted [14].

Principal component analysis (PCA), gene ontology (GO) analysis, and hierarchical clustering from the obtained normalized intensity data were performed using GeneSpring® software (Agilent). In GO analysis, GO terms were extracted if the p-value cut-off was inferior to 0.01. In hierarchical clustering, complete linkage and squared Euclidean distance were utilized as previously [15].

### 3.5 Gene network and upstream regulator analyses

In order to examine the gene network, the obtained data was analyzed using Ingenuity Pathways Analysis (IPA) tools (Ingenuity Systems, Mountain View, CA), a web-delivered application that enables the identification, visualization, and exploration of molecular interaction networks in gene expression data. In order to identify the potential upstream transcriptional regulators, an upstream regulator analysis, which can explain the observed gene expression changes in the obtained dataset, was performed using IPA tools. The top five upstream regulators were identified and the network containing the regulators and target genes was visualized to provide a hypothesis for gene regulation.

### 3.6 Quantitative PCR

Total RNA was subjected to reverse transcription with ReverTra Ace qPCR RT Master mix according to the manufacturer's instructions (Toyobo). The DNA samples were amplified with a CFX Connect Read-Time PCR Detection System (BioRad), THUNDERBIRD SYBR qPCR Mix (Toyobo), and the primer sets specific for human genes. The sequences of primer sets were as follows: forward primer, 5ʹ-CTGGAGTACTATGAGCGGGC-3ʹ and reverse primer, 5ʹ-TGGCTGATATCTGGGTGCCT-3ʹ for IFIT1; forward primer, 5ʹ-TGAGGAAGGGTGGACACAAC-3ʹ and reverse primer, 5ʹ-ACATCGCAATTGCCAGTCCA-3ʹ for IFIT3; forward primer, 5ʹ-CTGGATAGCAGCAGCCTCAG-3ʹ and reverse primer, 5ʹ-AGCTCCATAAGGAAGCACTCG-3ʹ for IRF7; forward primer, 5ʹ-TGGCATAACCAGAGTGGCTG-3ʹ and reverse primer, 5ʹ-CACCACCAGGCTGATTGTCT-3ʹ for Mx1; forward primer, 5ʹ-ACCATGCACTCTGTTTGCGA-3ʹ and reverse primer, 5ʹ-CGAAAGGCACCTATCCGTTC-3ʹ for TLR3; forward primer, 5ʹ-GCACCAACTACCCAGTGGAG-3ʹ and reverse primer, 5ʹ-TGGCGTCTGGTCTTTGACAG-3ʹ for TICAM1; forward primer, 5ʹ-AAGGCTGGGGCTCATTTGCA-3ʹ and reverse primer, 5ʹ-ATGACCTTGCCCACAGCCTT-3ʹ for GAPDH.

Each mRNA expression level was normalized with respect to the mRNA expression of GAPDH. Data are presented as means ± S.D. (n = 4).

## 4 Results and discussions

**Fig. 1:**
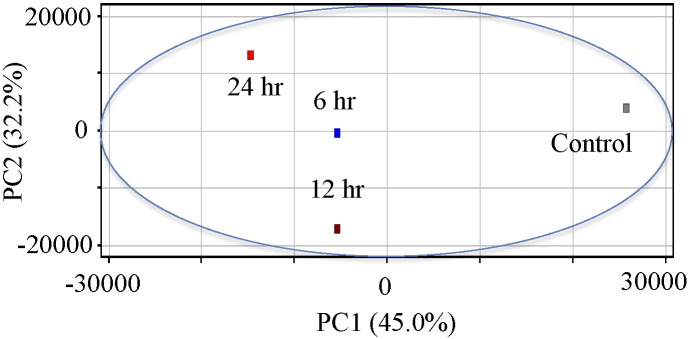
Principal component analysis (PCA) on the comprehensive gene expression analysis data. PCA was performed using Gene Spring software. HUVECs were X-irradiated by 2.5 Gy and then cultured for 6, 12, and 24 h until RNA extraction and followed by global gene expression analysis.

**Fig. 2:**
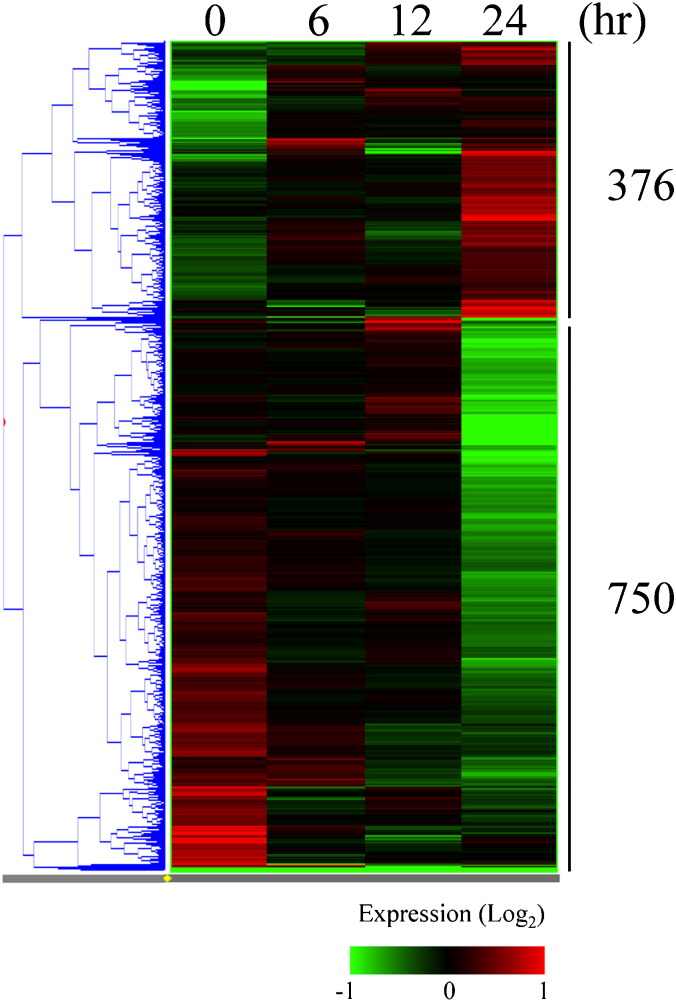
Gene expression profiles of non-irradiated and X-irradiated HUVECs. Genes that were differentially expressed by a factor of 1.5 or greater in HUVECs irradiated with 2.5 Gy X-ray are shown. Hierarchical clustering of differentially expressed genes was performed using the Gene Spring software.

HUVECs were irradiated with X-rays of 2.5 Gy and then were harvested 6, 12, and 24 h after irradiation for global transcriptomic analysis. After normalization of obtained intensities through the MAS5 algorithm, we performed PCA on gene expression data. The PCA revealed that the gene expression pattern in non-irradiated control cells was markedly distinct from that in irradiated cells, particularly in cells at 24 h after irradiation (Fig. 1). We identified 1126 probes that were differentially expressed by a factor of 1.5 or greater in either control cells or cells 24 h after irradiation. Hierarchical clustering of differentially expressed probes showed that the majority of 376 or 750 entities were gradually up- or down-regulated until 24 h (Fig. 2). In concordance with a previous study of ours [5], within 24 h after irradiation, the differentially expressed genes did not include NOS1-3 encoding neural NOS, inducible NOS, and eNOS, indicating that NO production after X-irradiation may be largely dependent on the post-transcriptional modification of eNOS, but independent of the transcriptional up-regulation of those NOS.

In the present study, we further performed a bioinformatics analysis to identify the gene ontology (GO) and the genetic network of differentially expressed genes to elucidate the radiation-induced inflammatory response except for NO production. From the GO analysis, we found that two of top three functions in down-regulated genes were involved in cell-cycle regulation (Supplementary Table 1). The down-regulated genes were associated with “Cell cycle” and “Cell cycle process,” which has been known as typical cellular response after X-irradiation. In line with the GO analysis, we previously reported that the expression of genes involved in the promotion of the cell cycle (e.g., CCNB1 and PLK1) were down-regulated in irradiated HeLa cells [9]. On the contrary, we found that all top three functions in up-regulated genes were involved in type-1-interferon responses (Table 1). The up-regulated genes were associated with “Response to type 1 interferon”, “type 1 interferon signaling pathway,” and “Cellular response to type 1 interferon”, which were not observed in the case of irradiated HeLa cells, indicating that the genes associated with the type-1-interferon pathway may contribute to the inflammation response in irradiated HUVEC cells.

**Fig. 3:**
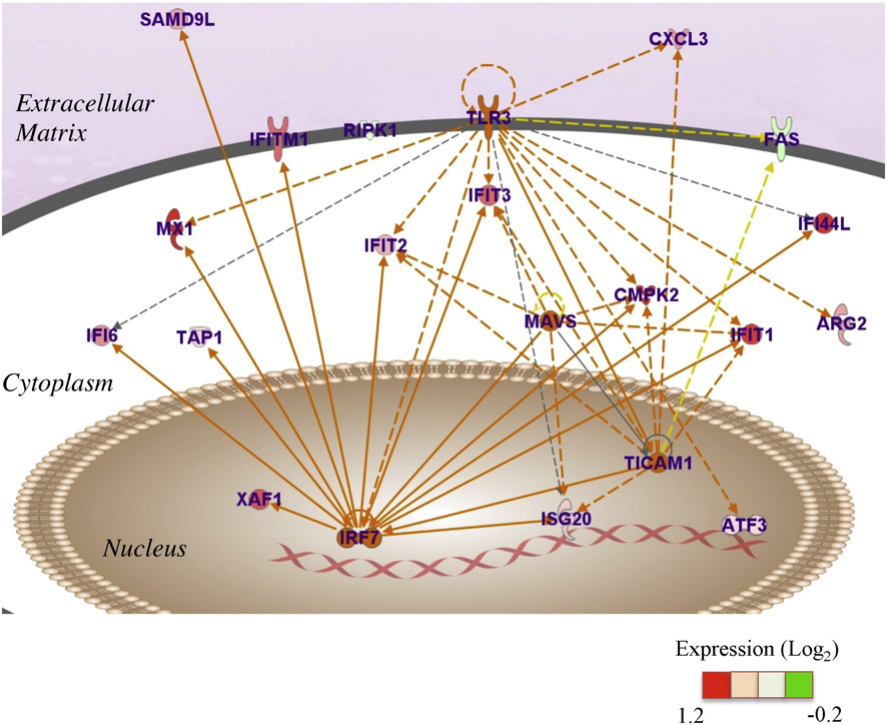
Gene network identified following gene expression analysis. Up-regulated genes after X-irradiation were analyzed using IPA tools. The network is displayed graphically as nodes (genes) and edges (biological relationships). The node color of the genes indicates the expression of genes. Solid lines and dashed lines indicate direct and indirect interaction between molecules, respectively.

In order to elucidate the interactions between the up-regulated genes, we performed a gene network analysis. The analysis identified a gene network containing interferon response factor 7 (IRF7) and its transcriptional targets (e.g., interferon-induced transcripts (IFITs) and Mx dynamin-like GTPase 1 (Mx1); Fig. 3 and Table 2), which were also listed in the top three GO.

The mammalian IRF family comprises nine members: IRF1-9 (reviewed in [16]). Among them, IRF7 is highly homologous with IRF3 and forms homodimer or heterodimer with IRF3 in order to show its transcriptional activity. IRF3 is constitutively expressed but IRF7 is strongly induced by type 1 interferon-mediated signaling. Mx1 and IFITs, called interferon stimulated genes (ISGs), are inflammatory mediators which can be transcriptionally up-regulated by IRF3 and IRF7 [17]. Mx1 and IFIT1 were reported to be strongly associated with carotid intima media thickness and coronary calcification.

Other ISGs such as IFIT3, interferon alpha-inducible protein 44 like (IFI44L), and IFI6, which were strongly expressed in endothelial cells from lupus patients [18], were also up-regulated in irradiated HUVECs. These genes were also identified as targets of IRF7 in a previous report [17]. It is unknown whether other transcriptional targets of IRF7, such as IFIT2, cytidine monophosphate kinase 2 (CMPK2), transporter associated with antigen processing 1 (TAP1), XIAP-associated factor 1 (XAF1), sterile alpha motif domain-containing protein 9 like (SAMD9L), and interferon-induced transmembrane protein 1 (IFITM1) [17], [19], are involved in cardiovascular disease. However, the activation of the IRF7 pathway seems to be one of the molecular inflammatory responses in irradiated HUVECs.

Specially, we found that Toll-like receptor 3 (TLR3), recently reported as a target of ionizing radiation [20], was a potential upstream gene in radiation-induced inflammatory response since the upstream analysis of genes using IPA tools identified TLR3 and TICAM1 as part of the top five upstream regulator genes (Table 3). A previous study demonstrated that the stimulation of TLR3 transcriptionally up-regulated and activated IRF7 [21], [22]. TICAM1 (also known as TRIF), a TLR3-associated molecule, was also part of the top five regulators and identified in the genetic network, indicating that the activation of the TLR3 pathway might be responsible for the up-regulation of ISGs. Interestingly, the transcription of TLR3 and TICAM1 was also slightly up-regulated after X-irradiation. One of the possible mechanisms underlying the transcriptional up-regulation of TLR3 is radiation-induced p53 signaling since a past study indicated that HCT116 cells harbor a functional p53 binding site on the promoter regions of TLR3 [23]. The detailed mechanism underlying the transcriptional regulation of TLR3 and TICAM1 by X-irradiation is still not clear. However, the up-regulation of these genes might result in the enhancement of TLR3-mediated signaling.

In addition to TLR3 and TICAM1, we found that the mitochondrial antiviral-signaling protein (MAVS) was listed as top five regulators. MAVS is considered to be important for ISG induction since ISG induction is not observed in myeloid dendritic cells with a defect in IRF3-IRF7, as well as in cells with a defect in MAVS [19]. Interestingly, MAVS transcription was also up-regulated in irradiated HUVECs, thus seemingly contributing to the induction of ISGs, even though the mechanism of MAVS transcription is not understood.

The up-regulation of TLR3, TICAM1, IRF7, IFIT1, IFIT3, and Mx1 at 24 h observed by microarray was further verified by quantitative real-time PCR analysis (Supplementary Fig. 1). The up-regulated genes and the gene network identified here may explain the inflammatory response induced by X-irradiation. In addition, the upstream analysis of up-regulated genes predicted that several genes in the identified network, such as IRF7 and its downstream molecules, might be regulated by TLR3, a molecule activated by a short fragment RNA released from irradiated cells [20]. Interestingly, we found that TLR3 and its component TICAM1 were transcriptionally up-regulated in response to irradiation, indicating that TLR3-IRF7-mediated inflammatory response pathway might be activated even by the transcription of TLR3 and associated molecule TICAM1 in response to X-irradiation.

The significance of the computational analysis should be elucidated in further biological studies. However, we expect our evidence to support further clinical studies uncovering the molecular basis of radiation-induced inflammatory response in HUVECs.

The following are the supplementary data related to this article.

Supplementary Table 1 Top three GO in down-regulated genes. Supplementary Table 1

Supplementary Fig. 1 Verification of microarray results by real-time qPCR. HUVEC cells were irradiated with 2.5 Gy IR and then cultured for 6, 12 and 24 h. Each expression level was normalized to GAPDH expression level. Data are presented as means. Supplementary Fig. 1

## Transparency document

Transparency document Image 2

## Figures and Tables

**Table 1:** Top three GO in up-regulated genes.

GO term	p-Value	Molecules
Response to type 1 interferon	5.71E-04	MX1, IFIT1, IFI6, ISG20, ISG15, PTPN6, IRF7, IFITM1, IFIT2, XAF1, IFIT3, TRIM56
Type 1 interferon signaling pathway	0.001	MX1, IFIT1, IFI6, ISG20, ISG15, PTPN6, IRF7, IFITM1, IFIT2, XAF1, IFIT3
Cellular response to type 1 interferon	0.001	MX1, IFIT1, IFI6, ISG20, ISG15, PTPN6, IRF7, IFITM1, IFIT2, XAF1, IFIT3

**Table 2:** Expression levels of genes in the identified genetic network.

Probe set ID	Gene symbol	Fold (cont. vs 24 h)	Cont. (RAW)	6 h (RAW)	12 h (RAW)	24 h (RAW)	Cont. (Log_2_)	6 h (Log_2_)	12 h (Log_2_)	24 h (Log_2_)
202086_at	MX1	2.5	252.4	286.3	246.8	629.3	− 0.09	0.09	− 0.12	1.23
202307_s_at	TAP1	1.6	569.8	861.9	858.7	918.4	− 0.59	0	0	0.09
202672_s_at	ATF3	2.1	152	282.3	299.2	321.3	− 0.93	− 0.04	0.04	0.14
203153_at	IFIT1	2.7	250.7	403.1	168.7	686.4	− 0.34	0.34	− 0.91	1.11
203945_at	ARG2	1.5	121.3	147.6	125.6	185.8	− 0.17	0.12	− 0.12	0.45
204415_at	IFI6	1.7	1314.2	1563.2	1417.6	2207.2	− 0.18	0.07	− 0.07	0.57
204439_at	IFI44L	2.4	151.4	185.9	112.9	370.4	− 0.15	0.15	− 0.57	1.14
204698_at	ISG20	1.5	260	370.1	302.1	401	− 0.36	0.15	− 0.15	0.26
206271_at	TLR3	1.8	83.1	103.9	108	149.5	− 0.35	− 0.03	0.03	0.5
207850_at	CXCL3	1.7	124.3	179.7	118.9	214.2	− 0.27	0.27	− 0.33	0.52
208436_s_at	IRF7	2	342.2	551.1	558.2	687	− 0.7	− 0.01	0.01	0.31
209941_at	RIPK1	1.9	78	152.2	162.3	149.4	− 0.95	0.01	0.11	− 0.01
213191_at	TICAM1	1.5	161.2	184.8	275.4	243.4	− 0.4	− 0.2	0.38	0.2
214022_s_at	IFITM1	1.8	636.9	716.4	526.9	1167.5	− 0.08	0.08	− 0.36	0.79
215719_x_at	FAS	1.5	313	620.3	510.1	475.6	− 0.65	0.33	0.05	− 0.05
226495_at	MAVS	1.7	1103.3	1340.1	1305.7	1821.3	− 0.26	0.02	− 0.02	0.46
226603_at	SAMD9L	1.5	511.9	549.7	613.7	788.5	− 0.18	− 0.08	0.08	0.44
226702_at	CMPK2	2.3	56.4	53.8	23.9	127.6	0.03	− 0.03	− 1.21	1.21
226757_at	IFIT2	2.4	59.8	129.6	97.4	146	− 0.91	0.21	− 0.21	0.38
228617_at	XAF1	2.1	269.9	269.3	321.5	560.3	− 0.13	− 0.13	0.13	0.93
229450_at	IFIT3	2.1	293.7	399.5	254.2	603.4	− 0.22	0.22	− 0.43	0.82

**Table 3:** Top five upstream regulators and target molecules.

Upstream regulator	Log ratio	Molecule type	Predicted state	p-Value	Target molecules in dataset
TLR3	0.847	Transmembrane receptor	Activated	7.92E-08	ARG2, ATF3, CMPK2,CXCL11, CXCL3, FAS, IFI44L, IFI6, IFIT1, IFIT2, IFIT3, IRF7, ISG15, ISG20, MX1, RIPK1, TICAM1, TLR3
MAVS	0.724	Other	Activated	2.54E-05	CMPK2, IFIT1, IFIT2, IFIT3, IRF7, ISG15, ISG20
IRF7	1.005	Transcription regulator	Activated	4.24E-07	CMPK2, IFI44L, IFI6, IFIT1, IFIT2, IFIT3, IFITM1, IRF7, ISG15, ISG20, MX1, SAMD9L, TAP1, XAF1
TICAM1	0.594	Other	Activated	1.60E-05	CMPK2, CXCL11, CXCL3, FAS, IFIT1, IFIT2, IFIT3, IRF7, ISG15, ISG20, TLR3
PPARD	1.165	Ligand-dependent nuclear receptor	Activated	1.87E-03	ACTA2, C10orf35, IFI44L, MMP9, PDE4C, PDPK1, PPARD, TCEA3, VLDLR
