# Supplementary material for: Comprehensive and computational analysis of genes in human umbilical vein endothelial cells responsive to X-irradiation
Source: Genom Data. 2016 May 16;8:126–30. doi: 10.1016/j.gdata.2016.05.007 (PMC4880795; doi:10.1016/j.gdata.2016.05.007)
Supplement: Supplementary Fig. 1 — Verification of microarray results by real-time qPCR. HUVEC cells were irradiated with 2.5 Gy IR and then cultured for 6, 12 and 24 h. Each expression level was normalized to GAPDH expression level. Data are presented as means. [file mmc2.pptx]

## Slide 1
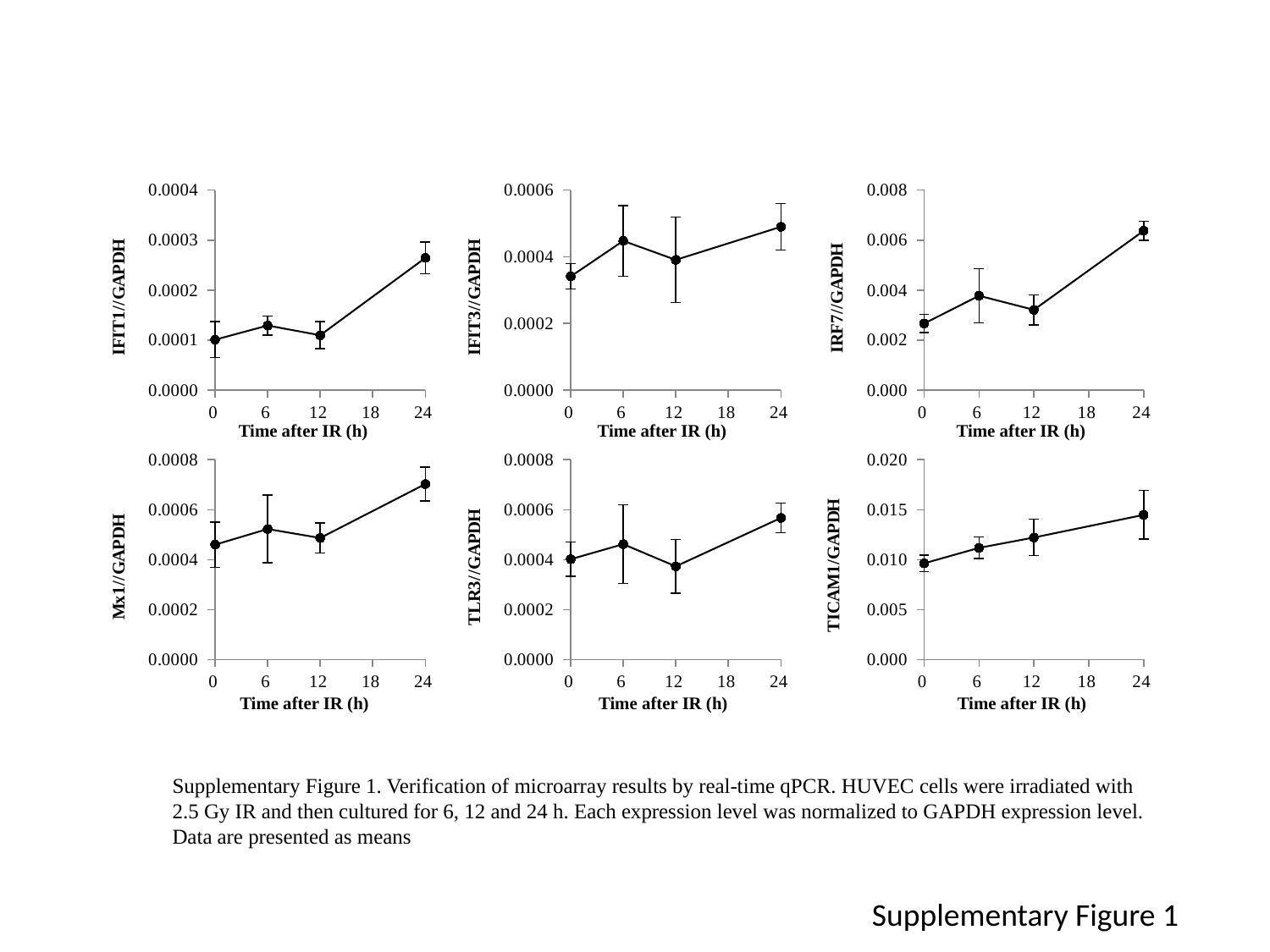

### Chart
| Category | IFIT1 |
|---|---|
### Chart
| Category | IFIT3_1 |
|---|---|
### Chart
| Category | IRF7_2 |
|---|---|Time after IR (h)
Time after IR (h)
Time after IR (h)
### Chart
| Category | Mx1 |
|---|---|
### Chart
| Category | TLR3 |
|---|---|
### Chart
| Category | TICAM1 |
|---|---|Time after IR (h)
Time after IR (h)
Time after IR (h)
Supplementary Figure 1. Verification of microarray results by real-time qPCR. HUVEC cells were irradiated with
2.5 Gy IR and then cultured for 6, 12 and 24 h. Each expression level was normalized to GAPDH expression level.
Data are presented as means
Supplementary Figure 1
